# VASCo: computation and visualization of annotated protein surface contacts

**DOI:** 10.1186/1471-2105-10-32

**Published:** 2009-01-24

**Authors:** Georg Steinkellner, Robert Rader, Gerhard G Thallinger, Christoph Kratky, Karl Gruber

**Affiliations:** 1 Institute of Molecular Biosciences, University of Graz, Humboldtstraße 50/3, 8010 Graz, Austria; 2 Research Centre Applied Biocatalysis, Petersgasse 14, 8010 Graz, Austria; 3 Institute for Genomics and Bioinformatics, Graz University of Technology, Petersgasse 14, 8010 Graz, Austria; 4 Christian Doppler Laboratory for Genomics and Bioinformatics, Petersgasse 14, 8010 Graz, Austria

## Abstract

**Background:**

Structural data from crystallographic analyses contain a vast amount of information on protein-protein contacts. Knowledge on protein-protein interactions is essential for understanding many processes in living cells. The methods to investigate these interactions range from genetics to biophysics, crystallography, bioinformatics and computer modeling. Also crystal contact information can be useful to understand biologically relevant protein oligomerisation as they rely in principle on the same physico-chemical interaction forces. Visualization of crystal and biological contact data including different surface properties can help to analyse protein-protein interactions.

**Results:**

VASCo is a program package for the calculation of protein surface properties and the visualization of annotated surfaces. Special emphasis is laid on protein-protein interactions, which are calculated based on surface point distances. The same approach is used to compare surfaces of two aligned molecules. Molecular properties such as electrostatic potential or hydrophobicity are mapped onto these surface points. Molecular surfaces and the corresponding properties are calculated using well established programs integrated into the package, as well as using custom developed programs. The modular package can easily be extended to include new properties for annotation. The output of the program is most conveniently displayed in PyMOL using a custom-made plug-in.

**Conclusion:**

VASCo supplements other available protein contact visualisation tools and provides additional information on biological interactions as well as on crystal contacts. The tool provides a unique feature to compare surfaces of two aligned molecules based on point distances and thereby facilitates the visualization and analysis of surface differences.

## Background

Knowledge on protein-protein interactions is essential for understanding many processes in living cells. These interactions are mediated through the respective molecular surfaces and are governed by the properties of the amino acid residues and atoms, which form these surfaces, as well as more distributed properties such as hydrophobicity and electrostatic potential. Structural data from crystallographic analyses deposited in the Protein Data Bank (PDB) [1,2] contain a vast amount of information on protein-protein contacts, including "biological" contacts which are also present in solution as well as contacts necessary for crystal formation. Several programs are available which allow the calculation and analysis of surface properties, e.g. to predict hotspots for protein interaction [3-5]. However, these programs are not designed to calculate, analyze and visualize actual protein-protein contact patches. Various reviews describe investigations about statistical analyses of protein-protein interaction, characterization of different interface properties [6-12] and the identification of contact patches especially focused on the distinction between biological interactions and crystal contacts [13-15]. Other studies describe specific properties used to discriminate between interfaces and non-interfaces like shape and geometric parameter complementarities, accessible surface comparison at multimerization as well as physicochemical properties, conservation scores and interface residue preferences and clusters [16]. Despite the fact that this information is very useful for the identification and analysis of biological contacts, it lacks a convenient visual representation of information especially for crystal contact surfaces and their properties. A plethora of macromolecular visualization tools exist, which are either web-based or stand alone programs (see the "The World Index of Molecular Visualization Resources" [17] for an overview). Most of them also provide structural analysis tools or are part of different databases which contain all sorts of organized structural annotation information like the GPSSServer [18], the Mark-Us server [19], the POLYVIEW-3D utility [20], or the program PocketPicker [21]. There are also databases which are mainly focused on protein contacts or interfaces like the SCOWLP [22]. This database is based on the SCOP [23] classification and provides interaction information on domain interfaces and uses Jmol [24] for visualization. Web based visualization tools like Jmol are very useful to give an overview of the structure including of the different provided information. However, they are less powerful than stand-alone programs like the Swiss-PDB Viewer [25] or PyMOL [26]. Not all of these tools provide sufficient surface representation features or the surface representation is generated on the fly. Consequently, the actual surface points are not accessible directly for annotation or calculation purposes. Software packages which do provide contact interaction information most often make use of atom to atom distances and atom coordinates instead of surface point coordinates. Many programs also do not take into account crystal symmetry. Therefore, we devised VASCo a program package enabling the annotation and visualization of surface properties and contact patches. Specifically we aimed at (i) identifying contact patches in protein crystal structures including contacts generated by crystal symmetry, (ii) annotating these patches according to different surface properties and (iii) analysing surface patches of proteins in contact with RNA, DNA or ligands. Additional aims were the convenient representation of annotated surfaces and the development of a distance calculation for the surface points in contact regions. For visualization we chose PyMOL because of its wide spread within the structural biology community and its broad functionality and expandability.

## Implementation

VASCo itself is a Python [27] based command line tool which makes use of the VASCo modules (also written in Python) to calculate properties and run external programs. The programs PatchCalc and HydroCalc, written in C, are used to calculate hydrophobicity as well as contact patches and distance values. The external programs MSMS [28] for surface calculation and DelPhi [29,30] for electrostatics calculations are separately available and licensed but included in the program package. The VASCo package integrates the calculation of molecular surfaces of proteins, the computation of different properties (such as hydrophobicity and electrostatic potential), the identification of contact patches, and a flexible visualization module. For the latter task we use the program PyMOL [26] representing molecular surfaces as "compiled graphic objects" (CGO), a PyMOL specific format allowing the generation of three-dimensional objects from building blocks such as spheres, cylinders and triangles. We developed a plug-in, which reads the VASCo surface file and generates CGOs based on the provided information. This Phython-based plug-in provides a convenient interface to visualize the surface output.

## Results and discussion

VASCo is a program package for the calculation of protein surface properties and the visualization of annotated surfaces. Our software uses a unique surface point based approach where each of these points can be directly annotated by different properties. The surfaces and interaction patches are visualized in PyMOL using a custom-made plug-in.

Surface points are defined by the solvent excluded surface (SES) of a protein. In addition, it identifies contact patches between protein molecules based on a point distance cutoff, considering also symmetry equivalent molecules in a crystal. Thus, surface points are separated into contact and non-contact areas allowing separate analysis. The current set of properties contains the electrostatic potential, the hydrophobicity and the contact distance and is easily extendable due to the modular structure of the program package. The different modules and programs are integrated into an analysis pipeline to allow fast and efficient analysis of the protein structures. Special emphasis was given to the visualization of crystal contact patches and surfaces which is especially important for the analysis of this kind of data. We do not distinguish between biological and crystal contact patches automatically, yet the patch information with the mapped properties may help to differentiate them visually. Our software can serve as a supplement to other available visualisation tools and provides additional information on protein-protein contacts which are relevant for structural biologists and crystallographers as well.

Another strength of the VASCo program is the possibility to compare molecular surfaces of biomolecules. To that end, a PDB file has to be generated containing two superimposed structures (*e.g*. a homology model and its template, two homologues from different organisms or an apo- protein with its substrate bound form.). By neglecting the symmetry information one is able to annotate surfaces with a surface difference value which corresponds to the minimum distance of a particular surface point in one molecule to any surface point in the other molecule. These calculations can be used to identify regions on those surfaces which differ significantly from each other or to investigate the influence of mutations on surface shape.

### Calculation Pipeline

**Figure 1:**
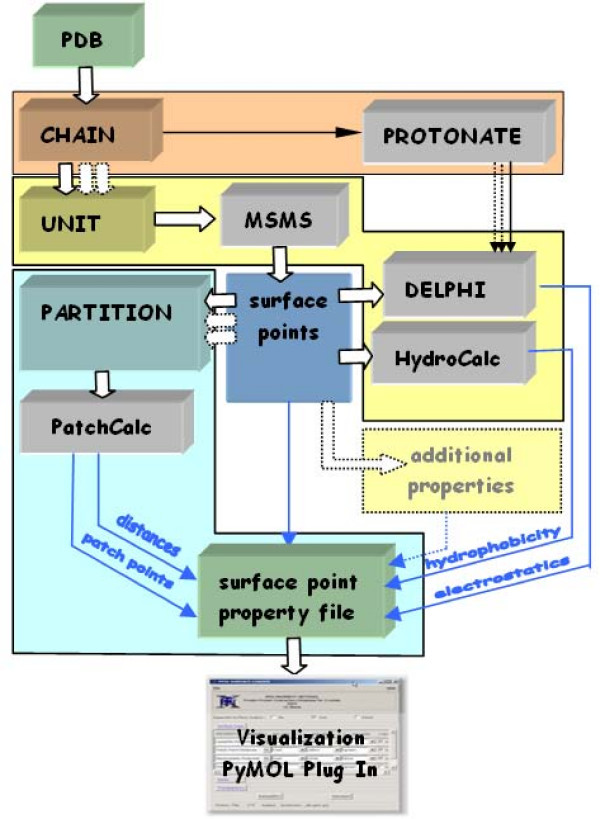
**Overview of the VASCo pipeline**. The chain, the unit and the partition sections are marked with corresponding colours (red for chain, yellow for unit and green for partition sections). Gray boxes represent programs; green boxes indicate input and output files. Blue arrows represent the flow of the different calculated properties. White arrows show the main program path, whereas dotted arrows indicate "many-to-one" relationships within the pipeline.

The Python based command line program called VASCo.py, reads a PDB file (or a list of PDB files for the analysis of a larger number of structures) and in turn calls the various modules and programs performing the necessary calculations (Fig. 1). The output of the program is a GZIP-compressed text file containing information on the surface itself (coordinates of surface vertices, list of triangles) as well as on the calculated properties, which can be visualized in PyMOL using the VASCo surface viewer plug-in (see below). Reasonable default parameters (especially for DelPhi) and libraries (such as HC values, atomic radii, partial charges, and space group symmetry) are provided, but can easily be changed for particular purposes.

### Surface determination

Molecular surface points are determined by MSMS (Michel Sanner's Molecular Surface) version 2.5.5 [28] using the SES (solvent excluded surface) definition, a probe radius of 1.4 Å and a vertex density of 1.0. There are other surface calculation programs available including NACCESS [31], Surface Racer [32], ASC [33,34], or the Molecular Surface Package [35]. We chose the program MSMS because it provides surface point files with additional information such as triangulation and normal vectors which can be used directly for visualization.

### Hydrophobicity

The command line program HydroCalc was developed to calculate hydrophobicity values at each surface point. A library of atomic hydrophobic contribution values (HC) was created based on the values derived by Ghose *et al*. [36] and newer calculations of Viswanadhan and Ghose *et al*. [37,38]. These HC values [see Additional file 1] can be seen as fragmental increments (f_i_) to the total lipophilicity of the molecule. Andry et al. have created a distance dependent function for a so called molecular lipophilicity potential (MLP) [39] the applicability of which has been proven for small molecules. Due to its unsuitability for large molecules another form of the MLP definition was used [40] (formula 1).

$$MLP=\frac{\sum_{i=1}^{N}f_{i}\cdot g(d_{i})}{\sum_{i=1}^{N}g(d_{i})}$$

Formula 1: Molecular lipophilicity potential (MLP). f_i _is the partial lipophilicity of the i-th fragment of a molecule. d_i _is the distance of the surface point from the center of the fragment i. N is the amount of fragments considered for the calculation and g(d_i_) is the distance function for the i-th fragment.

The program uses a Fermi-type distance function [40] (formula 2) to calculate the contribution of a particular atom to the molecular lipophilicity potential (MLP) at a certain surface point. The empirical drop-off parameters C_1 _and C_2 _were set to standard values (C_1_= 1 Å^-1^, C_2 _= 4 Å). The Fermi-function simulates the decrease of hydrophobic influence of distant atoms to avoid false assignments within protein molecules.

$$g(d)=\frac{e^{-C_{1}C_{2}}+1}{e^{C_{1}(d-C_{2})}+1}$$

Formula 2: Fermi-type distance function. C_1 _and C_2 _are empirical drop-off parameters. d is the distance of a certain surface point from the center of the fragment.

Compared to other algorithms to assign hydrophobicity values to surface points, this approach has the advantage that the hydrophobicity calculation can be carried out with distance dependent atomic contributions on every surface point separately. This is in contrast to other strategies where whole amino acid hydrophobicity scores are used and mapped onto the surface [41]. Our calculation is clearly more time consuming but has the advantage that the hydrophobicity is smoothly distributed over the surface. Due to its distance dependent character it accounts for the three dimensional arrangement of the atoms and their contributions to the hydrophobicity on each surface point.

### Electrostatic potential

The program DelPhi [29,30] is used to calculate the electrostatic potential at the molecular surface points. As default parameters we used a grid spacing of 1 Å with the macromolecule taking up 60% of the calculation box. Internal and external dielectric constants were set to 4 and 80 respectively. An ion exclusion radius of 2 Å and a salt concentration of 0.145 mol l^-1 ^were applied. The probe radius for the surface calculation is the same as used for MSMS (1.4 Å). All parameters can be changed by the user, if necessary. DelPhi requires the positions of (selected) hydrogen atoms. As most of the structure files deposited in the PDB miss this information, we calculate hydrogen atom positions using a modified version of the program Protonate, which is part of the AMBER program package [42]. We consider only backbone and N-terminal hydrogen atoms and assign charges only to fully charged amino acids as well as backbone atoms. Histidines are assigned a total charge of +0.5. The DelPhi output file contains the electrostatic potential at the given coordinates (surface point coordinates produced by MSMS) in units of *kT*/e (1 *kT*/e = 25.6 mV/e = 0.593 kcal/mol/e), where *k *is the Boltzmann-constant and the temperature *T *is set to 300 K.

### Contact patch calculation

The program PatchCalc was developed to calculate interaction patches based on a distance cutoff involving surface points. This includes interactions between different unit surfaces as well as interactions between symmetry related surface points. We define a "unit" as an assembly of protein chains (plus heterocomponents) for which surface points and corresponding properties will be calculated. By default each protein chain forms a separate unit, but one can combine several chains to a larger unit, *e.g*. to an oligomer. The combination of all thus defined units forms the so called "partition". The command line program PatchCalc calculates all the contact patches of each unit within a partition (also including crystal symmetry). A surface point contact is assumed, when the distance between surface points is below a certain threshold, which is 1.5 Å by default but can be changed by the user. In order to utilize crystal symmetry the program requires information on the unit cell (to calculate fractional coordinates) and on the space group, which is both provided automatically within the VASCo-program. Atomic cartesian coordinates are transformed to fractional coordinates [43]. Space group symmetry is provided as library of transformation matrices and vectors (converted from data available in the CCP4 package [44]) which are applied to the fractional coordinates. Nearest neighbors of a particular surface point are identified taking translational symmetry into account. The final output contains the point to point distance as well as unit and symmetry information which are used to annotate the different contact patches.

### Visualization

**Figure 2:**
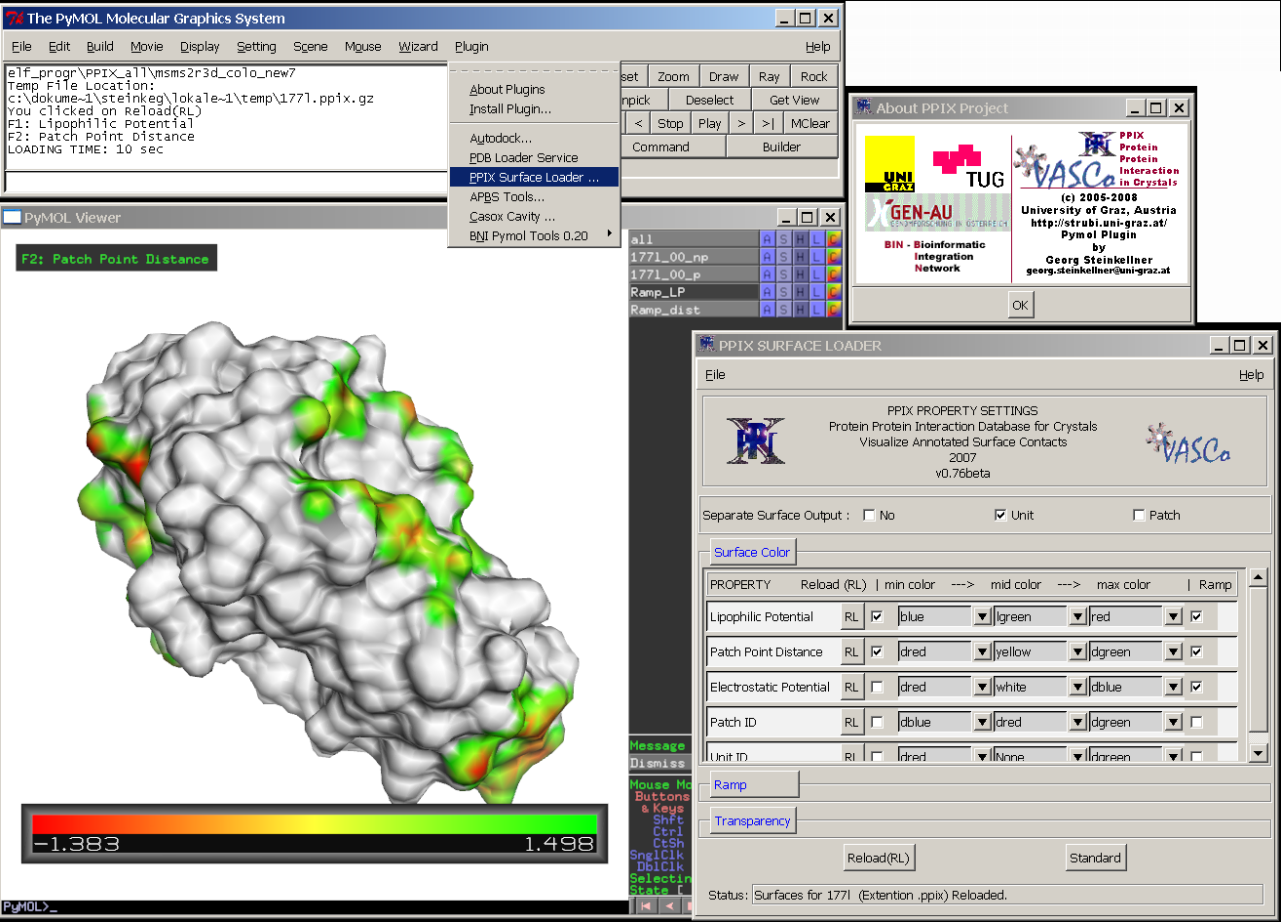
**Example of visualization of VASCo results loaded with PyMOL plug-in**. Molecular surfaces represented by PyMOL-CGOs. Patches representing contacts to symmetry equivalent molecules are coloured according to contact distance values. The loader is integrated in the plug-in dropdown menu of PyMOL. Dependent on the input surface file different properties can be shown and analyzed. Once the selected properties of interest are loaded one can switch easily between them using the associated function keys

A PyMOL plug-in was developed for displaying the surface properties calculated by the VASCo program. Each identified surface contact patch can be viewed and analyzed separately. The user can freely define colours and transparency levels of the different surfaces. The minimum and maximum values for a colour ramp describing a certain property are calculated automatically, but can as well be set manually (Fig. 2). To speed up loading of the surface representation the user can select to view just those annotated surfaces, which are the current focus of interest. After loading of the surfaces one can switch fast and easily between the annotated surfaces by using the standard function keys. Because PyMOL's internal ray-tracing routine also works for the additional properties calculated by the program package, it can be used to create high-resolution, publication quality figures. The plug-in configures itself depending on the available surface properties in the input file and is therefore flexible to show different properties depending on the currently active surface file. This feature will be especially useful when new surface properties will be provided in later versions of the VASCo program package.

## Examples

### Surface property visualization

**Figure 3:**
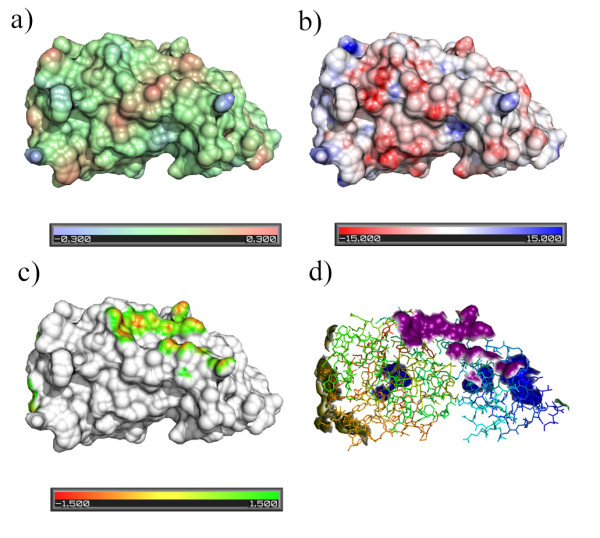
**Visualization of VASCo results for PDB-entry **1Y6I. Molecular surfaces represented by PyMOL-CGOs and coloured according to hydrophobicity (a), electrostatic potential (b). Patches representing contacts to symmetry equivalent molecules coloured according to contact distance values (c) and according to patch type (d). This figure was generated using the VASCo PyMOL plug-in.

The VASCo program can be used to visualize surface interaction patches between different units (defined by chains) as well as to analyse crystal contact patches calculated with crystal symmetry information. The structure of a GUN4 homolog [PDB:1Y6I] [45] was chosen as an example. The asymmetric unit of this structure consists of a single polypeptide chain with a length of 233 amino acid residues. As the structure comprises only one chain, this chain forms the only unit. Figure 3 shows the molecular surface coloured according to hydrophobicity (3a) and electrostatic potential (3b) employing the default colour ramp. Figures 3c and 3d contain information on interactions with symmetry related molecules (space group P2_1_2_1_2_1_). Interaction patches are coloured according to contact distances (Fig. 3c) and to patch types (Fig. 3d). The latter are defined by the interacting unit and the applied symmetry operation.

**Figure 4:**
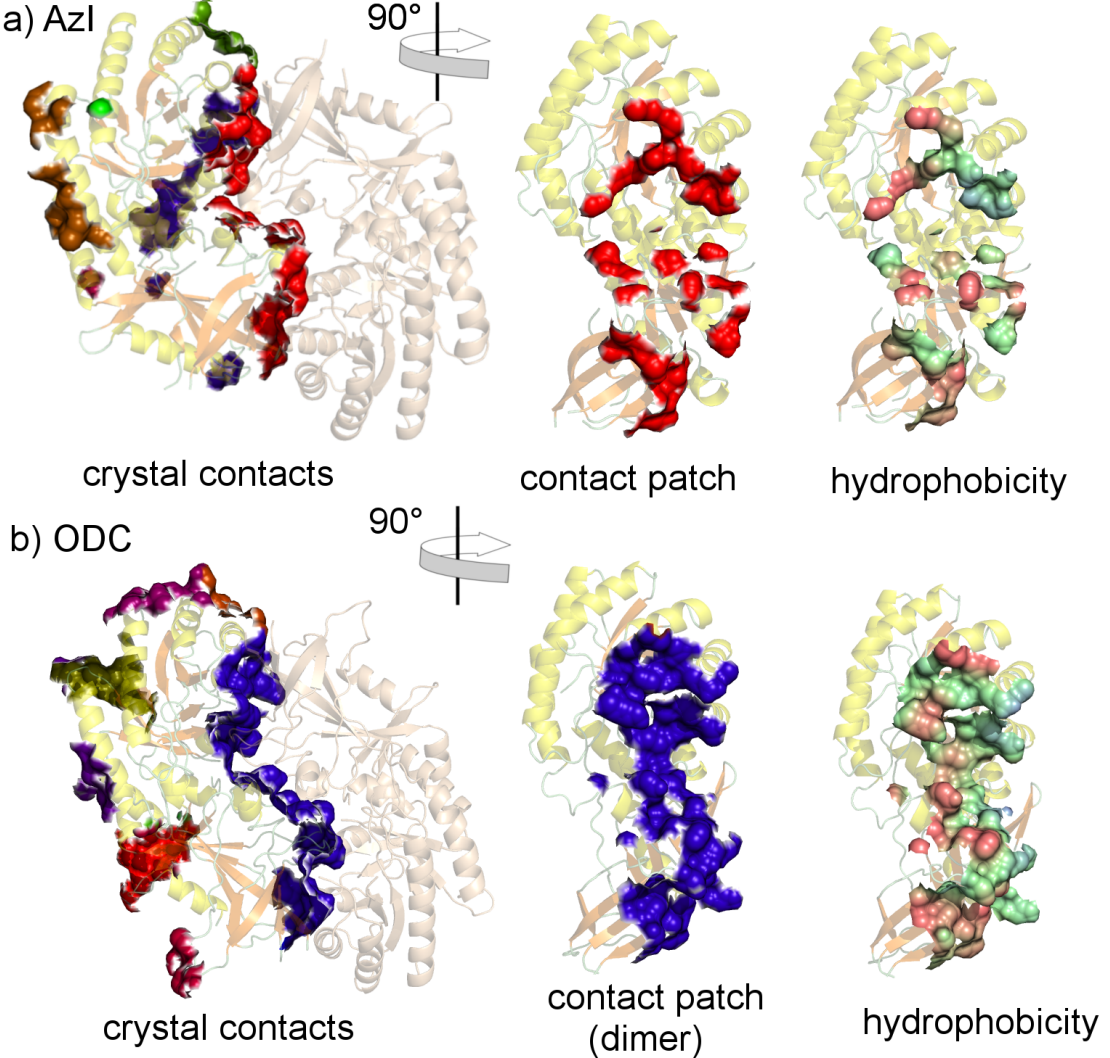
**Comparison of the dimer interfaces of antizyme inhibitor (AzI) and orithine decarboxylase (ODC)**. Surface contact patches generated with VASCo. a) AzI crystal contact patches and dimer interface patch (red). b) ODC interface including crystal contact patches for chain A and dimer interface patch (blue). On the right site the interface patches are coloured by hydrophobicity.

Another example of the results of contact patch calculation is shown in Figure 4. The antizyme inhibitor (AzI) which regulates cellular polyamine homestasis is compared with the highly homologous orithine decarboxilase (ODC) which is in contrast to AzI enzymatically active [46]. Figures 4a and 4b show all contact patches for chain A of AzI [PDB:3PTN] [46] and chain A of ODC [PDB:7ODC] [47], respectively. In the case of ODC two protein chains are present in the asymmetric unit, but a very similar "dimer" of AzI is generated by crystal symmetry (Fig. 4). A visual comparison of the proposed dimer interfaces reveals a significantly smaller surface contact size in the case of AzI (Fig. 4a -red surface patch) as well as a more compact contact surface in the case of ODC (Fig. 4b – blue surface patch). This analysis suggests that this interface in AzI could be an artefact of crystal formation and nonphysiological. Biochemical studies indeed showed that AzI exists as a monomer in solution while ODC is dimeric [46]. In addition, the "hydrophobic zipper" [46] can easily be seen on the ODC dimer interface (Fig. 4b – hydrophobicity) which is missing in case of the AzI (Fig. 4a – hydrophobicity). Thus, VASCo can be very helpful in getting a first visual impression of the interfaces and their properties.

### Visualization of surface differences

**Figure 5:**
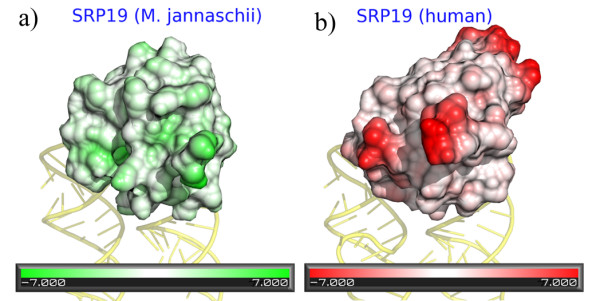
**Structural differences of SRP19 from *M. jannaschii *(a) and *H sapiens *(b)**. The surfaces are coloured according to VASCo-surface difference values. The colour ramp goes from white (no difference) to either green or red (+/- 7 Å).

The comparison of molecular surfaces of biomolecules is an additional strength of the VASCo program. A specific example of a surface comparison involving two homologous RNA binding proteins is shown in Figure 5 highlighting surface differences between signal recognition particle 19 (SRP19) from *H. sapiens *[48] and *Methanococcus jannaschii *[49].

**Figure 6:**
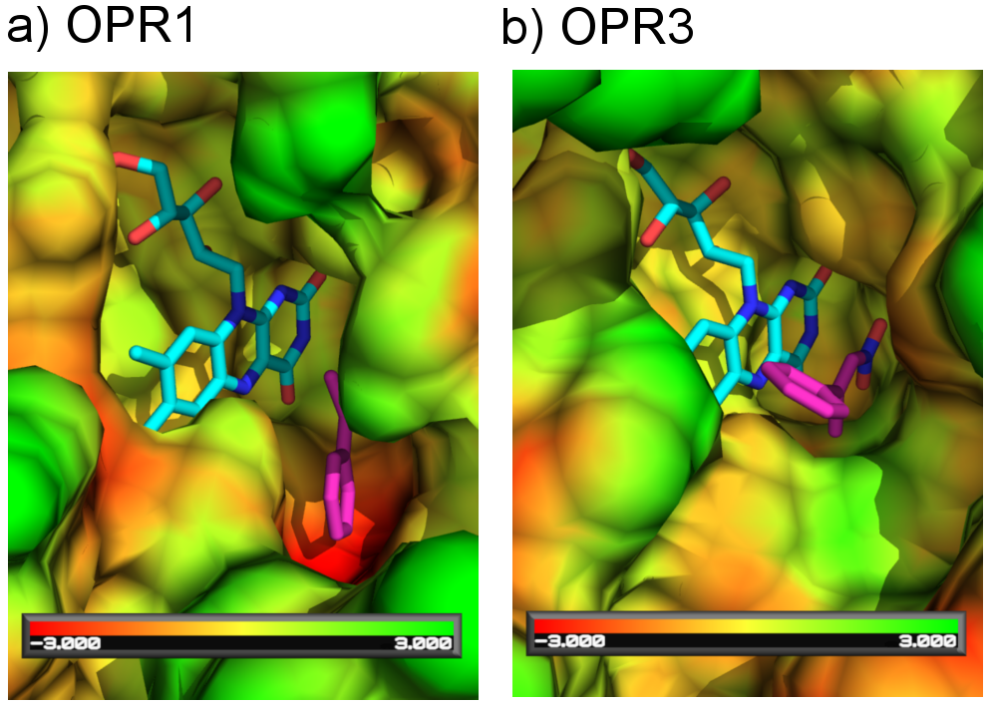
**Surface difference comparison of the active site of two enoate reductases**. The active site surfaces are coloured according to surface difference values (red -3 Å, green +3 Å, yellow no difference). a) OPR1 active site surface difference to OPR3. b) OPR3 active site surface difference to OPR1.

Another example shows the active site of two old yellow enzyme (OYE) homologues (Fig. 6). These two enzymes (OPR1 and OPR3) exhibit opposite enantioselectivity in the reduction of a nitroalkene substrate, which is supposed to be caused by subtle structural differences [50]. The differences in the surface topography around the active sites of OPR1 [PDB:1ICP] [51] and OPR3 [PDB:2HSA] [52] are shown in Figure 6 by mapping minimum point distances onto the respective molecular surfaces. In this example, red areas indicate surface indentations which are not present in the other molecule, whereas green areas indicate convexities. Specifically, the red area close to the flavin cofactor in OPR1 emphasizes a deep pocket missing in OPR3, which, however, is important for the proper binding of the substrate (Fig. 6a). The lack of this pocket in OPR3 necessitates a different binding mode of the substrate thereby yielding the opposite enantiomer. Our tools provide a convenient way to visualize such differences and to analyze their biological significance.

## Conclusion

The VASCo package provides convenient tools for the representation of annotated surfaces. It allows the facile inclusion of new properties (such as conservation scores) for surface mapping. The calculated surface is always divided into patch and non-patch surfaces allowing separate visual analysis of these regions. The tool also provides a unique point distance approach for the analysis and visualization of surface differences between two structures. By using the common protein representation and visualization tool PyMOL as environment for the plug-in, the annotated surfaces can be visualized. The plug-in automatically accommodates additional surface properties provided in the input file. We expect that VASCo will expand and grow over time especially by integrating new surface properties and property calculations.

## Availability and requirements

Project name: VASCo

Project home page: .

Operating systems: Windows, Unix

Programming Languages: Python, C

Software packages: Python 2.4 or higher , PyMOL 0.95 or higher 

Hardware requirements: Processor: 3 GHz Pentium 4 or similar, Memory: 1 GB RAM Video Card: 3D OpenGL compatible graphics accelerator card with 256 MB RAM

License: The VASCo program is free for academic use but includes third party programs like the MSMS program for surface calculation and the DelPhi v. 4.0 program for electrostatic calculations which have to be registered (free of charge for academic use). For more information about licensing see 

Any restrictions to non-academics:

If you are interested in a commercial use license for VASCo, please send your name, address, fax and telephone numbers and email address to: VASCo@genome.tugraz.at. Commercial versions of MSMS and DelPhi have to be obtained from .Michel F. Sanner and .Raquel Norel respectively.

## Authors' contributions

GS designed and implemented the software and drafted the manuscript. RR was involved in software design and testing. GGT contributed to the draft of the manuscript, webpage creation and coordination of software packaging and licensing. KG participated in the design and implementation of HydroCalc and PatchCalc and helped to draft the manuscript. General scientific advice was given by CK and KG. All authors read and approved the final manuscript.

## Supplementary Material

Additional file 1 **Calculated HC values for amino acids.** The data table provided contains the calculated hydrophobic contribution values for the standard amino acids. Click here for file
