# Supplementary material for: VASCo: computation and visualization of annotated protein surface contacts
Source: BMC Bioinformatics. 2009 Jan 24;10:32. doi: 10.1186/1471-2105-10-32 (PMC2649047; doi:10.1186/1471-2105-10-32)
Supplement: Additional file 1 — Calculated HC values for amino acids. The data table provided contains the calculated hydrophobic contribution values for the standard amino acids. [file 1471-2105-10-32-S1.pdf]

Calculated HC values for amino acids

Table 1 Hydrophobic contribution of standard amino acids calculated with different atom type values[2,3,4]

amino acid 3 letter code	atom name	MSMS-radius	Type#[1,2]	(1) HC value 1986[2]	(2) HC value 1989[3]	(3) HC value 1998[4]
ALA	N	1.8	72+50	-0.2289	-0.3788	-0.6149
	CA	2.37	8+2x51	-0.4621	-0.1012	0.3663
	CB	2.17	1+3x46	0.6594	0.6483	0.6420
	C	1.7	40	-0.1703	0.0709	-0.1002
	O	1.6	58	-0.1729	-0.3514	-0.0233
ARG	N	1.8	72+50	-0.2289	-0.3788	-0.6149
	CA	2.37	8+2x51	-0.4621	-0.1012	0.3663
	CB	2.23	2+2x46	0.4616	0.3963	0.4562
	CG	2.23	2+2x46	0.4616	0.3963	0.4562
	CD	2.23	6+2x46	-0.0867	0.0466	0.2196
	NE	1.6	67+50	-0.0886	-0.0455	-0.4204
	CZ	2.1	44	-0.2692	0.1847	0.1388
	NH1	1.6	(66+67+50)/2+50	-0.2817	-0.2894	-0.5851
	NH2	1.6	(66+67+50)/2+50	-0.2817	-0.2894	-0.5851
	C	1.7	40	-0.1703	0.0709	-0.1002
	O	1.6	58	-0.1729	-0.3514	-0.0233
ASN	N	1.8	72+50	-0.2289	-0.3788	-0.6149
	CA	2.37	8+2x51	-0.4621	-0.1012	0.3663
	CB	2.23	2+2x46	0.4616	0.3963	0.4562
	CG	2.1	40	-0.1703	0.0709	-0.1002
	OD1	1.6	58	-0.1729	-0.3514	-0.0233
	ND2	1.6	72+2x50	-0.5992	-0.7048	-0.7185
	C	1.7	40	-0.1703	0.0709	-0.1002
	O	1.6	58	-0.1729	-0.3514	-0.0233
ASP	N	1.8	72+50	-0.2289	-0.3788	-0.6149
	CA	2.37	8+2x51	-0.4621	-0.1012	0.3663
	CB	2.23	2+2x46	0.4616	0.3963	0.4562
	CG	2.1	40	-0.1703	0.0709	-0.1002
	OD1	1.6	(57+58)/2+50	-0.1962	-0.2587	-0.1216
	OD2	1.6	(57+58)/2+50	-0.1962	-0.2587	-0.1216
	C	1.7	40	-0.1703	0.0709	-0.1002
	O	1.6	58	-0.1729	-0.3514	-0.0233
CYS	N	1.8	72+50	-0.2289	-0.3788	-0.6149
	CA	2.37	8+2x51	-0.4621	-0.1012	0.3663
	C	1.7	40	-0.1703	0.0709	-0.1002
	O	1.6	58	-0.1729	-0.3514	-0.0233
	CB	2.23	6+2x46	-0.0867	0.0466	0.2196
	SG	1.89	106+50	0.6449	0.4008	0.5110
GLU	N	1.8	72+50	-0.2289	-0.3788	-0.6149
	CA	2.37	8+2x51	-0.4621	-0.1012	0.3663
	CB	2.23	2+2x46	0.4616	0.3963	0.4562
	CG	2.23	2+2x46	0.4616	0.3963	0.4562

amino acid 3 letter code	atom name	MSMS-radius	Type#[1,2]	(1) HC value 1986[2]	(2) HC value 1989[3]	(3) HC value 1998[4]
	CD	2.1	40	-0.1703	0.0709	-0.1002
	OE1	1.6	(57+58)/2+50	-0.1962	-0.2587	-0.1216
	OE2	1.6	(57+58)/2+50	-0.1962	-0.2587	-0.1216
	C	1.7	40	-0.1703	0.0709	-0.1002
	O	1.6	58	-0.1729	-0.3514	-0.0233
GLN	N	1.8	72+50	-0.2289	-0.3788	-0.6149
	CA	2.37	8+2x51	-0.4621	-0.1012	0.3663
	CB	2.23	2+2x46	0.4616	0.3963	0.4562
	CG	2.23	2+2x46	0.4616	0.3963	0.4562
	CD	2.1	40	-0.1703	0.0709	-0.1002
	OE1	1.6	58	-0.1729	-0.3514	-0.0233
	NE2	1.6	72+2x50	-0.5992	-0.7048	-0.7185
	C	1.7	40	-0.1703	0.0709	-0.1002
	O	1.6	58	-0.1729	-0.3514	-0.0233
GLY	N	1.8	72+50	-0.2289	-0.3788	-0.6149
	CA	2.23	8+2x51	-0.4621	-0.1012	0.3663
	C	1.7	40	-0.1703	0.0709	-0.1002
	O	1.6	58	-0.1729	-0.3514	-0.0233
HIS	N	1.8	72+50	-0.2289	-0.3788	-0.6149
	CA	2.37	8+2x51	-0.4621	-0.1012	0.3663
	CB	2.23	2+2x46	0.4616	0.3963	0.4562
	CG	2.1	25	0.3345	0.1600	0.1492
	CD2	2.1	33+47	0.3952	0.1569	0.2578
	ND1	1.6	73+50	-0.0210	0.0938	0.0223
	CE1	2.1	42+47	-0.3509	0.2027	0.4154
	NE2	1.6	73	0.3493	0.4198	0.1259
	C	1.7	40	-0.1703	0.0709	-0.1002
	O	1.6	58	-0.1729	-0.3514	-0.0233
ILE	N	1.8	72+50	-0.2289	-0.3788	-0.6149
	CA	2.37	8+2x51	-0.4621	-0.1012	0.3663
	CB	2.37	3+46	0.1514	0.0785	0.0660
	CG2	2.17	1+3x46	0.6594	0.6483	0.6420
	CG1	2.23	2+2x46	0.4616	0.3963	0.4562
	CD1	2.17	1+3x46	0.6594	0.6483	0.6420
	C	1.7	40	-0.1703	0.0709	-0.1002
	O	1.6	58	-0.1729	-0.3514	-0.0233
LEU	N	1.8	72+50	-0.2289	-0.3788	-0.6149
	CA	2.37	8+2x51	-0.4621	-0.1012	0.3663
	CB	2.23	2+2x46	0.4616	0.3963	0.4562
	CG	2.37	3+46	0.1514	0.0785	0.0660
	CD1	2.17	1+3x46	0.6594	0.6483	0.6420
	CD2	2.17	1+3x46	0.6594	0.6483	0.6420
	C	1.7	40	-0.1703	0.0709	-0.1002
	O	1.6	58	-0.1729	-0.3514	-0.0233
LYS	N	1.8	72+50	-0.2289	-0.3788	-0.6149
	CA	2.37	8+2x51	-0.4621	-0.1012	0.3663
	CB	2.23	2+2x46	0.4616	0.3963	0.4562
	CG	2.23	2+2x46	0.4616	0.3963	0.4562
	CD	2.23	2+2x46	0.4616	0.3963	0.4562

amino acid 3 letter code	atom name	MSMS-radius	Type#[1,2]	(1) HC value 1986[2]	(2) HC value 1989[3]	(3) HC value 1998[4]
	CE	2.23	6+2x46	-0.0867	0.0466	0.2196
	NZ	1.6	66+2x50	-0.4748	-0.5333	-0.7499
	C	1.7	40	-0.1703	0.0709	-0.1002
	O	1.6	58	-0.1729	-0.3514	-0.0233
MET	N	1.8	72+50	-0.2289	-0.3788	-0.6149
	CA	2.37	8+2x51	-0.4621	-0.1012	0.3663
	CB	2.23	2+2x46	0.4616	0.3963	0.4562
	CG	2.23	6+2x46	-0.0867	0.0466	0.2196
	SD	1.89	107	1.0339	0.6145	0.5906
	CE	2.17	5+3x46	0.1460	0.2430	0.4143
	C	1.7	40	-0.1703	0.0709	-0.1002
	O	1.6	58	-0.1729	-0.3514	-0.0233
PHE	N	1.8	72+50	-0.2289	-0.3788	-0.6149
	CA	2.37	8+2x51	-0.4621	-0.1012	0.3663
	CB	2.23	2+2x46	0.4616	0.3963	0.4562
	CG	2.1	25	0.3345	0.1600	0.1492
	CD1	2.1	24+47	0.3174	0.3411	0.3050
	CD2	2.1	24+47	0.3174	0.3411	0.3050
	CE1	2.1	24+47	0.3174	0.3411	0.3050
	CE2	2.1	24+47	0.3174	0.3411	0.3050
	CZ	2.1	24+47	0.3174	0.3411	0.3050
	C	1.7	40	-0.1703	0.0709	-0.1002
	O	1.6	58	-0.1729	-0.3514	-0.0233
PRO	N	1.8	68	0.3990	0.3954	0.0132
	CA	2.37	8+2x51	-0.4621	-0.1012	0.3663
	CB	2.23	2+2x46	0.4616	0.3963	0.4562
	CG	2.23	2+2x46	0.4616	0.3963	0.4562
	CD	2.23	6+2x46	-0.0867	0.0466	0.2196
	C	1.7	40	-0.1703	0.0709	-0.1002
	O	1.6	58	-0.1729	-0.3514	-0.0233
SER	N	1.8	72+50	-0.2289	-0.3788	-0.6149
	CA	2.37	8+2x51	-0.4621	-0.1012	0.3663
	CB	2.23	6+2x46	-0.0867	0.0466	0.2196
	OG	1.6	56+50	-0.4220	-0.1858	-0.4603
	C	1.7	40	-0.1703	0.0709	-0.1002
	O	1.6	58	-0.1729	-0.3514	-0.0233
THR	N	1.8	72+50	-0.2289	-0.3788	-0.6149
	CA	2.37	8+2x51	-0.4621	-0.1012	0.3663
	CB	2.37	8+46	-0.5156	-0.0792	0.0536
	OG1	1.6	56+50	-0.4220	-0.1858	-0.4603
	CG2	2.17	1+3x46	0.6594	0.6483	0.6420
	C	1.7	40	-0.1703	0.0709	-0.1002
	O	1.6	58	-0.1729	-0.3514	-0.0233
TRP	N	1.8	72+50	-0.2289	-0.3788	-0.6149
	CA	2.37	8+2x51	-0.4621	-0.1012	0.3663
	CB	2.23	2+2x46	0.4616	0.3963	0.4562
	CG	2.1	25	0.3345	0.1600	0.1492
	CD2	2.1	25	0.3345	0.1600	0.1492
	CE2	2.1	34	0.2455	-0.2782	0.2813

amino acid 3 letter code	atom name	MSMS-radius	Type#[1,2]	(1) HC value 1986[2]	(2) HC value 1989[3]	(3) HC value 1998[4]
	CE3	2.1	24+47	0.3147	0.3411	0.3050
	CD1	2.1	24+47	0.3174	0.3411	0.3050
	NE1	1.6	75+50	-0.1946	-0.4366	-0.2660
	CZ2	2.1	24+47	0.3174	0.3411	0.3050
	CZ3	2.1	24+47	0.3174	0.3411	0.3050
	CH2	2.1	24+47	0.3174	0.3411	0.3050
	C	1.7	40	-0.1703	0.0709	-0.1002
	O	1.6	58	-0.1729	-0.3514	-0.0233
TYR	N	1.8	72+50	-0.2289	-0.3788	-0.6149
	CA	2.37	8+2x51	-0.4621	-0.1012	0.3663
	CB	2.23	2+2x46	0.4616	0.3963	0.4562
	CG	2.1	25	0.3345	0.1600	0.1492
	CD1	2.1	24+47	0.3174	0.3411	0.3050
	CE1	2.1	24+47	0.3174	0.3411	0.3050
	CD2	2.1	24+47	0.3174	0.3411	0.3050
	CE2	2.1	24+47	0.3174	0.3411	0.3050
	CZ	2.1	26	-0.1153	-0.1033	0.1539
	OH	1.6	57+50	0.1509	0.1600	-0.1163
	C	1.7	40	-0.1703	0.0709	-0.1002
	O	1.6	58	-0.1729	-0.3514	-0.0233
VAL	N	1.8	72+50	-0.2289	-0.3788	-0.6149
	CA	2.37	8+2x51	-0.4621	-0.1012	0.3663
	CB	2.37	3+46	0.1514	0.0785	0.0660
	CG1	2.17	1+3x46	0.6594	0.6483	0.6420
	CG2	2.17	1+3x46	0.6594	0.6483	0.6420
	C	1.7	40	-0.1703	0.0709	-0.1002
	O	1.6	58	-0.1729	-0.3514	-0.0233

1. Sauer, O., *Gases for Structure Determination in Protein Crystallography*, in *Institute of Chemistry, Structural Biology*. PhD Thesis 2001, Karl Franzens University of Graz: Graz. p. 208.

2. Ghose, A.K. and G.M. Crippen, *Atomic physicochemical parameters for three-dimensional structure-directed quantitative structure-activity relationships. I. Partition coefficients as a measure of hydrophobicity*. Journal of Computational Chemistry, 1986. **7**(4): p. 565-77.

3. Viswanadhan, V.N., et al., *Atomic physicochemical parameters for three dimensional structure directed quantitative structure-activity relationships. 4. Additional parameters for hydrophobic and dispersive interactions and their application for an automated superposition of certain naturally occurring nucleoside antibiotics.* Journal of Chemical Information and Computer Sciences, 1989. **29**(3): p. 163-72.

4. Ghose, A.K., V.N. Viswanadhan, and J.J. Wendoloski, *Prediction of Hydrophobic (Lipophilic) Properties of Small Organic Molecules Using Fragmental Methods: An Analysis of ALOGP and CLOGP Methods.* Journal of Physical Chemistry A, 1998. **102**(21): p. 3762-3772.
